# The role of pain self-efficacy and pain catastrophising in the relationship between chronic pain and depression: A moderated mediation model

**DOI:** 10.1371/journal.pone.0303775

**Published:** 2024-05-23

**Authors:** Lauren Kardash, Cindy Lee Wall, Mal Flack, Amelia Searle

**Affiliations:** 1 Faculty of Health, Charles Darwin University, Darwin, NT, Australia; 2 College of Medicine and Public Health, Flinders University, Bedford Park, SA, Australia; 3 Pain Management Unit, Flinders Medical Centre, Bedford Park, SA, Australia; Iran University of Medical Sciences, ISLAMIC REPUBLIC OF IRAN

## Abstract

Chronic pain is a substantial health problem with a high prevalence of comorbid depression. To understand the link between chronic pain and depression, cognitive factors including pain catastrophising and pain self-efficacy have been theorised as significant contributing variables. There is relatively strong evidence that pain catastrophising mediates the relationship between pain severity and depression symptoms. There is also emerging evidence that the mediation role of pain catastrophising may vary as a function of pain self-efficacy. However, it is unknown whether this model will apply in a tertiary pain clinic sample. Thus, this study aimed to examine the respective moderating and mediating roles of pain self-efficacy and pain catastrophising on the association between pain severity and depressive symptoms in a large clinical sample of Australian adults living with chronic pain. Participants (n = 1195) completed all questionnaire measures prior to their first appointments at one tertiary pain service. As expected, the PROCESS path analysis showed that pain catastrophising mediated the relationship between pain severity and depressive symptoms. Further, there was support for the moderating effect of pain self-efficacy; as pain self-efficacy decreased, the relationship strengthened between both pain severity and pain catastrophising, as well as pain catastrophising and depressive symptoms. These findings may have important clinical implications including how relationships between these factors may be considered in the provision of care for those with chronic pain. Notably, these measures could be used in triaging processes to inform treatment decisions.

## Introduction

Chronic pain is persistent non-cancer pain that lasts longer than three months or past the expected time for healing [1]. Chronic pain is a significant health problem, currently estimated to affect one in five Australian adults [2]. Further, people with chronic pain are likely to experience associated impaired functioning and mental health problems [2]. Comorbid depression rates are particularly high amongst Australians with chronic pain, with 40% of patients attending pain management units reporting being severely or extremely severely depressed [3,4]. In addition, only 57% of individuals who complete an episode of care with a pain management service report a clinically significant improvement in depression [4]. Cognitive factors including pain catastrophising and pain self-efficacy are considered likely contributors to depression severity in people with chronic pain [5–8], although further evaluation is needed to investigate their specific roles. Thus, the aim of this study was to examine the role of pain catastrophising and pain self-efficacy in the relationship between pain severity and depressive symptoms.

### Chronic pain and depression

The positive relationship between chronic pain and depression has been demonstrated extensively [9–12]. This is commonly accepted as a reciprocal relationship, with longitudinal research demonstrating increases in one variable being associated with increases in the other [13]. The importance of understanding the link between pain and depression is evident in findings that chronic pain patients with depression often have greater impairment and poorer treatment outcomes [5,6,14,15]. For instance, people with chronic pain who also experience depression, have a lower quality of life and greater impairment than people without depression [5,15,16]. Also, treatment outcomes can be jeopardized when chronic pain and depression are both present, but treatment is only targeted towards one condition [5,6,17]. For example, individuals being treated specifically for chronic pain who also have depressive symptoms, are more likely to experience poorer treatment outcomes such as higher pain severity, poorer quality of life, and functional impairment [5,17,18]. Similarly, poorer responses to mood interventions are seen for people being treated for depression who are also experiencing pain [5,6]. The level of depression severity in those with chronic pain has been shown to have important healthcare implications, with moderate and severe levels of depression being significantly associated with higher total healthcare use independent of pain [19]. This suggests there are potential healthcare benefits in reducing the level of depression in individuals with chronic pain. Given the significant prevalence of chronic pain and comorbid depression, and the associated poorer outcomes and health burden, understanding the relationship between depression and chronic pain is essential. This includes identifying modifiable factors that protect against or contribute to the maintenance of depression.

### Pain catastrophising

Cognitive factors contribute to depressive symptoms in individuals with chronic pain [8,20,21]. One of these cognitive factors is pain catastrophising, which is described as a tendency to exaggerate negative judgment of pain sensation, to ruminate, and feel helpless about the pain experience [20]. Pain catastrophising is associated with depression in those with chronic pain [22,23]. Researchers have demonstrated that pain catastrophising mediates the relationship between pain severity and depressive symptoms in individuals with chronic pain [10,12,24]. For instance, Sánchez-Rodrígue et al. [12] found that pain catastrophising mediated the relationship between pain severity and depressive symptoms in a sample recruited from the baseline of a multi-component pain and depression management program [12]. A two-wave longitudinal study by Wood et al. [10] further examined this mediation effect by measuring changes in pain catastrophising and depressive symptoms over time in a sample of 141 older adults with chronic non-cancer pain. Findings showed that changes in pain catastrophising completely mediated the relationship between pain severity and depressive symptoms over six months [10].

### Pain self-efficacy

Another cognitive factor that has been suggested to contribute to depression in individuals with chronic pain is pain self-efficacy. In general, self-efficacy is conceptualised as an individual’s confidence to perform tasks in the face of obstacles and aversive experiences, in turn influencing the level of effort expended and the length of time they persist [25]. In the context of people experiencing chronic pain, self-efficacy beliefs are expected to include not only the expectation that an individual could complete a certain task but also their confidence to persist despite their pain [26]. It has been argued that people with higher pain self-efficacy may be more likely to utilize their skills and available resources to persevere in managing their pain [27,28].

Pain self-efficacy has been associated with levels of depression in chronic pain populations [21,29,30]. Studies have found that people with higher pain self-efficacy are likely to have fewer depressive symptoms compared to those with low pain self-efficacy [21,30]. Pain self-efficacy has also been tested as a mediator of the relationship between chronic pain severity and depression. For example, in a cross-sectional study of 70 community members with spinal cord injury, Craig et al. [31] found that self-efficacy partially mediated the relationship between pain severity and depressed mood, reducing depressive symptoms when self-efficacy was high. Likewise, Miró et al. [32], found that self-efficacy mediated the relationship between pain intensity and depression in women with Fibromyalgia. In a more recent study, Schumann et al. [24] investigated the effects of a 3-week interdisciplinary pain rehabilitation program on outcomes including depression. Results demonstrated that an increase in pain self-efficacy and a decrease in pain catastrophising were associated with a reduction in depression [24]. Interestingly, changes in pain self-efficacy over the pre-treatment to follow-up period appeared more impactful [24]. This may potentially reflect the benefits of increasing pain self-efficacy in producing long-term change by reducing engagement in maladaptive responses. Similarly, Pjanic et al. [33] found evidence of self-efficacy moderating the relationship between pain severity and depression after one year.

### Pain self-efficacy as a moderator

Limited studies have examined the influence of pain self-efficacy as a moderator in the relationships between chronic pain, pain catastrophising and depression. Conceptualized as a protective factor, pain self-efficacy is expected to lead to pain-related challenges being perceived as obstacles to be overcome, persistence after setbacks, and utilization of coping strategies [26]. Therefore, those with higher pain self-efficacy may be less likely to engage in catastrophic thinking such as ruminating and feeling helpless about the pain sensation. Recently, Cheng et al. [8] tested a model of chronic pain that integrated pain self-efficacy and pain catastrophising. Specifically, pain self-efficacy was hypothesised to moderate the relationship between pain severity and pain catastrophising, with pain catastrophising as a predecessor of depressive symptoms [8]. A community sample of Chinese older adults (aged over 60) with chronic pain were recruited to complete a survey. Results supported the moderated mediation model, indicating that the positive relationship between pain severity on depressive symptoms via the mediator of pain catastrophising was reduced for individuals with higher levels of pain self-efficacy. Additionally, findings demonstrated that pain severity remained directly associated with depressive symptoms in this model, although the strength of this relationship was reduced as pain self-efficacy increased. This study appears to be the only study to have shown that pain self-efficacy moderated the effects of pain catastrophising in a mediation model. Notably, this community sample presented with only moderate levels of pain, depressive symptoms, pain catastrophising, and high levels of pain self-efficacy.

### The current study

The aim of the current study is to assess how the cognitive factors of pain catastrophising and pain self-efficacy are associated with pain severity and depressive symptoms in a large Australian chronic pain clinical sample. Specifically, we aim to test the model proposed by Cheng et al. [8] in a sample of adults who typically present with higher levels of pain severity, depressive symptoms, and pain catastrophising than adults in the general community. In addition, the current study will adapt Cheng et al.’s model by testing whether pain self-efficacy also moderates the relationship between pain catastrophising and depressive symptoms. That is, we argue that if individuals are engaging in catastrophic thinking about their pain, then depression severity may be reduced by strengthening the confidence to persist in adaptive strategies despite this. Identifying the points at which pain self-efficacy may reduce the strength of the relationship between pain severity and depression could inform clinical interventions.

Based on previous research, several hypotheses are proposed. First, it is predicted that pain severity is positively associated with depressive symptoms and pain catastrophising. Second, that pain catastrophising mediates the relationship between pain severity and depressive symptoms. Third, pain self-efficacy moderates the relationship between pain severity and depressive symptoms. That is, pain severity will have a stronger direct relationship with depressive symptoms when pain self-efficacy is low than when pain self-efficacy is high. Fourth, pain self-efficacy will moderate the relationship between pain severity and pain catastrophising. That is, pain severity will have a stronger relationship with pain catastrophising when pain self-efficacy is low. Finally, pain self-efficacy will also moderate the relationship between pain catastrophising and depressive symptoms.

## Methods

### Participants and procedure

The participants were Australian adults referred by specialist medical care practitioners to a Level 1 Tertiary Pain Service. All participants were experiencing severe chronic non-cancer pain, consistent with ICD-11 diagnostic criteria [1]. Participants were drawn from historical, de-identified data collected at the Flinders Medical Centre Pain Management Unit in South Australia, Australia. All participant information was collected at their initial referral or presentation to the Pain Unit (baseline) from January 2019 to March 2022. The measures used in this study are currently used as part of standard clinical practice in the Pain Unit and are collected in collaboration with the National Electronic Persistent Pain Outcomes Collaboration (ePPOC) [34]. ePPOC is a national benchmarking system aimed at improving services and outcomes for individuals accessing pain management services. While 1519 participants had completed the measures, 324 participants were removed after screening for missing data. The final sample included 1195 participants that were mostly female (females = 790; males = 402; sex not stated = 3) and had a mean age of 52.69 years (SD = 14.27; range = 20–90). In relation to pain duration, 65.4% of participants had experienced their pain for over 5 years, 20.4% had a duration of pain between 2 to 5 years, 9.4% had a pain duration between 12 months to 2 years, and 4.9% had experienced their pain between 3 to 12 months. Due to the nature of the referrals to the Pain Management Unit (e.g., a free public hospital-based service), no participants had unsettled insurance claims.

This research was approved by The Southern Adelaide Clinical Human Research Ethics Committee (SAC HREC) and the SALHN Manager, Governance and Ethics (LNR/22/SAC/8). A waiver of consent was granted as there was no foreseeable risk of harm or discomfort to participants due to the use of existing de-identified secondary data. All data were fully anonymized before transfer to the researchers. Reciprocal Approval was also obtained from the Charles Darwin University Human Research Ethics Committee.

## Measures

### Depressive symptoms

To assess the severity of depressive symptoms, the depression subscale of the Depression Anxiety Stress Scale (Short version) (DASS-21) was used [35]. The depression subscale consists of 7 items and asked participants to rate the applicability of each item to them over the past week on a 4-point Likert scale (0 = *never* to 3 = *almost always*). Sample items are "I felt that life is meaningless" and "I felt down-hearted and blue". Participant scores are calculated as a total score, with a higher score representing more severe depressive symptoms. Score ranges of 0–4, 5–6, 7–10, 11–13, and 14–21 indicate normal, mild, moderate, severe, and extremely severe depressive symptoms respectively [35]. The DASS21 depression subscale demonstrated good internal consistency (α = .94) and has been shown to display good discriminant and convergent validity [36]. Additionally, multiple studies have demonstrated construct validity for the subscale in chronic pain populations [37,38].

### Pain severity

The pain intensity subscale of the Brief Pain Inventory (BPI) was used to measure participants’ pain severity [39]. The pain intensity subscale consists of 4 items and asked participants to rate their pain experience on average, worst, and least pain over the last week, and their current pain. This is completed on an 11-point Likert scale from 0 = *no pain* to 10 = *pain as bad as you can imagine*. Sample items are "your pain at its worst in the last week" and "your pain at its least in the last week". Scores are calculated as an average score, with higher scores representing greater pain severity on average. Score ranges of 0–4, 5–6, and 7–10 indicate mild, moderate, and severe pain respectively [39]. The BPI has been demonstrated as having good psychometric properties. It has been shown to have good construct and concurrent predictive validity in different populations, including those experiencing chronic non-cancer pain [39,40]. The pain intensity subscale of the BPI demonstrated good internal consistency (α = .85).

### Pain catastrophising

The Pain Catastrophising Scale (PCS) was used to assess pain catastrophising (α = .950) [41]. The PCS consists of 13 items assessing three domains including rumination, magnification and helplessness. Participants were required to rate the degree to which they experienced catastrophic thoughts on a 5-point Likert scale (0 = *not at all* to 4 = *all the time*). Sample items are "I worry all the time about whether the pain will end" and "I keep thinking about how much it hurts". Participant scores are calculated as total scores, with higher scores representing greater levels of pain catastrophising. Score ranges of 0–20, 20–30, and 30–52 indicate mild, high and severe levels of pain catastrophising respectively [41]. The PCS demonstrates high test-retest reliability across a 6-week period, and adequate construct and concurrent validity [41,42]. The PCS displayed good internal consistency, with a Cronbach’s α of .95.

### Pain self-efficacy

The Pain Self-Efficacy Questionnaire (PSEQ) was used to assess participants’ level of confidence to function, despite their pain [26]. The PSEQ consists of 10 items and asked participants to rate their confidence to do a range of activities despite their pain on a 7-point Likert scale (0 = *not at all confident* to 6 = *completely confident*). Sample items are "I can enjoy things, despite the pain" and "I can cope with my pain in most situations". Participant scores are calculated as total scores, with higher scores indicating stronger pain self-efficacy beliefs which are associated with lower pain-related disability. Score ranges of 0–19, 20–30, 31–40, 41–60 indicate severe, moderate, mild, and minimal pain-related impairment [43]. There is good evidence for the validity and reliability of the PSEQ which demonstrates high test-retest reliability across a 3-month period, strong construct validity, and good concurrent validity [26]. The PSEQ demonstrated good internal consistency (α = .93).

### Covariates

Background information including participant sex, age, and pain duration (in years) were included as covariates in the analysis to control for their potential effects.

## Data analysis

Data analyses were performed using SPSS [44]. First, descriptive analyses were conducted to explore variable characteristics. This was followed by Pearson correlation analyses to examine the associations amongst the variables of pain severity, pain catastrophising, pain self-efficacy, and depressive symptoms. The proposed moderated mediation model was tested using the SPSS PROCESS macro [45], which examines both the mediating and moderating effects in a single model. Predefined model 59 from the PROCESS macro (version 4.1) was used to test the moderated mediation model, in which the a path (pain severity to pain catastrophising), the b path (pain catastrophising to depressive symptoms), and the c’ path (pain severity to depressive symptoms) are all moderated (by pain self-efficacy). Further, the significance of an indirect or direct effect can vary at different values of the moderator. These conditional interaction effects were examined using the Johnson-Neyman technique. The variables of pain severity, pain catastrophising, and pain self-efficacy were mean centred to increase effect interpretability [45,46], and the analysis was based on 5,000 bootstrapped samples, using 95% percentile confidence intervals.

The main model variables were screened for normality, linearity, homoscedasticity, multicollinearity, singularity, and for univariate and multivariate outliers. Scores were normally distributed, with skewness scores ranging from -.115 to .558 and kurtosis from -1.055 to .073. Sex, age, and pain duration were included in the model as covariates, consistent with previous research [e.g., 8,12]. Four participants showed evidence of multivariate outliers, with Mahalanobis distances >20. However, these were not excluded from analysis due to all cases having a Cook’s Distance under 1, indicating no significant influence on the model parameters [47].

## Results

### Descriptive data and correlations

Descriptive statistics and bivariate correlations amongst the relevant variables are presented in Table 1. The mean score of pain severity was 6.20 (SD = 1.65), suggesting participants on average experienced between moderate to severe pain. Specifically, 20.33% of participants reported experiencing mild pain, 44.94% reported experiencing moderate pain, and 34.73% reported experiencing severe pain. The mean score for depressive symptoms was 9.13 (SD = 6.32), with 29.87% of participants reporting depressive symptoms within normal range, 10.71% reporting mild depressive symptoms, 20.59% reporting moderate depressive symptoms, 10.96% reported severe depressive symptoms, and 27.87% reported extremely severe depressive symptoms. Participants on average indicated experiencing high levels of pain catastrophising with a mean score of 26.90 (SD = 13.63). 33.56% of participants reported mild pain catastrophising, 25.77% reported high pain catastrophising, and 40.67% reported severe levels of pain catastrophising. Pain self-efficacy was moderately low, with participants’ mean score of 21.06 (SD = 12.65). Notably, 49.54% reported severely low pain self-efficacy, 29.04% reported moderately low pain self-efficacy, 13.31% reported mildly low pain self-efficacy, and 8.12% reported pain self-efficacy with minimal associated pain-related disability. Notably, the characteristics of this study’s participant sample are generally consistent with the typical, national specialist pain service demographic profile [4].

**Table 1:** Correlations and descriptive statistics.

Variable	1	2	3	4	5	M	SD
1. Pain Severity	-					6.20	1.65
2. Pain Catastrophising	.41**	-				26.90	13.63
3. Pain Self-efficacy	-38**	-.48**	-			21.06	12.65
4. Depressive symptoms	.30**	.66**	-.50**	-		9.13	6.32
5. Pain Duration	.05**	.01	.08**	.07*	-	4.46	0.85
6. Age	.06*	-.09**	-.05	-.05	.08**	52.69	14.27
7. Sex	.08**	-.05	-.01	-.07*	-.03	-	-

Note: *p < .05

**p < .01; bivariate associations between sex other constructs are point-biserial correlation coefficients.

Pain severity showed small-moderate positive correlations with both depressive symptoms (*r* = .30, *p* < .001) and pain catastrophising (*r* = .41, *p* < .001). Pain catastrophising also showed a large positive correlation with depressive symptoms (*r* = .66, *p* < .001). Pain self-efficacy was moderately-largely negatively correlated with pain severity (*r* = -.38, *p* < .001), depressive symptoms (*r* = -.50, *p* < .001), and pain catastrophising (*r* = -.48, *p* < .001).

**Fig 1:**
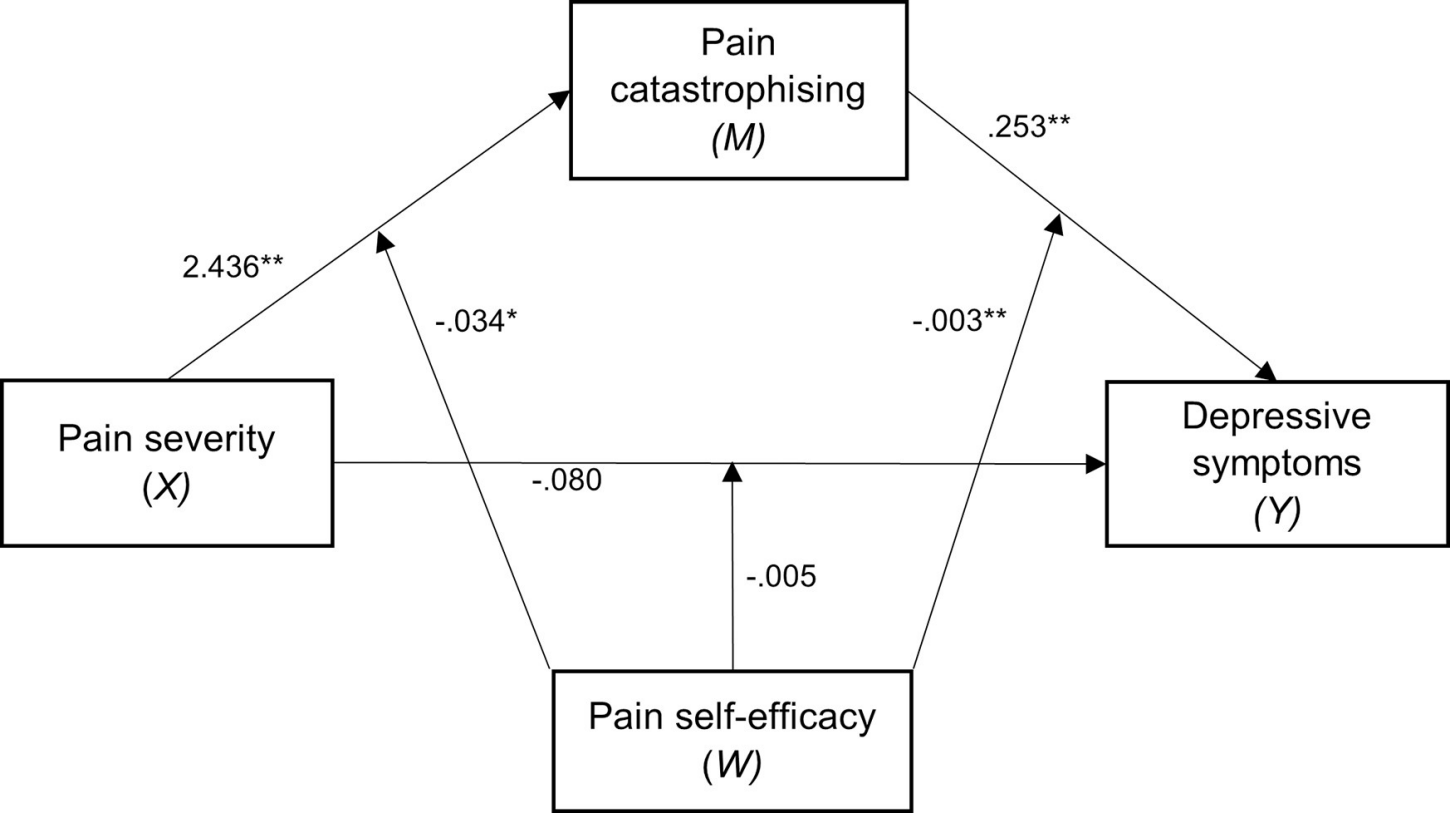
Moderated mediation model controlling for covariates of age, sex, and pain duration. *Note*. All coefficients are unstandardized (*p < .05, **p < .01). Covariates are omitted to enhance the clarity of the model.

The tested moderated mediation model, which includes the covariates of age, sex, and pain duration, is depicted in Fig 1. Relevant statistics for the moderated mediation analysis are presented in Table 2. The direct effect between pain severity and depressive symptoms (β = -.080, *CI* [-0.258, 0.098]), and the moderating effect of pain self-efficacy on this path (β = -.005, *CI* [-0.017, 0.008]) were both small and non-significant. Sex and age were not associated with the depressive symptoms, although sex (female) and age were negatively associated with pain catastrophising.

**Table 2:** Testing moderated mediation of pain self-efficacy and pain catastrophising on depressive symptoms.

Predictors	*On Pain Catastrophising (mediator)*				*On Depressive symptoms (outcome variable)*			
	β	SE	*p*	*95% CI*	β	SE	*p*	*95% CI*
Sex	-2.439	0.701	.005	[-3.815, -1.063]	-0.536	0.279	.055	[-1.083, 0.011]
Age	-0.086	0.023	.003	[-0.132, -0.040]	0.005	0.009	.596	[-0.013, 0.023]
Pain Duration	-0.120	0.388	.756	[-0.882, 0.641]	0.4394	0.154	.010	[0.093, 0.696]
Pain Severity	2.436	0.218	< .001	[2.009, 2.841]	-0.080	0.091	.379	[-0.258, 0.098]
Pain Self-efficacy	-0.398	0.028	< .001	[-0.453, -0.342]	-0.131	0.012	< .001	[-0.155, -0.107]
Pain Catastrophising	-	-	-	-	0.253	0.012	< .001	[0.231, 0.276]
Pain Severity x Pain Self-efficacy	-0.034	0.014	.015	[-0.062, -0.007]	-0.005	0.006	.454	[-0.017, 0.008]
Pain Catastrophising x Pain Self-efficacy	-	-	-	-	-0.003	0.002	< .001	[-0.005, -0.001]
R^2^	0.310		< .001		0.500		< .001	
F	88.577				147.687			

Note: Analysis was conducted using Hayes’ PROCESS model 59.

There was a significant indirect effect between pain severity and depressive symptoms via the mediator of pain catastrophising (β = .618, *CI* [.497, .741]). Further, this mediation effect was significantly moderated by pain self-efficacy, including both on the relationship between pain severity and pain catastrophising (β = -.034, *CI* [-.061, -.007]), and the relationship between pain catastrophising and depressive symptoms (β = -.003, *CI* [-.005, -.001]). Specifically, as pain self-efficacy decreased, the indirect effect of pain severity on depressive symptoms via pain catastrophising increased. The conditional indirect effect estimates are shown in Table 3.

**Table 3:** Direct and indirect effects on depressive symptoms, controlling for sex, age and pain duration.

			95% CI	
Pain Severity → Depressive symptoms	*B*	SE *B*	Lower Bound	Upper Bound
Indirect effect at severely low PSE (-1SD)	0.934	.091	0.654	1.012
Indirect effect at moderately low PSE (Mean)	0.618	.063	0.497	0.741
Indirect effect at mildly low PSE (+1SD)	0.433	.077	0.286	0.587
Direct effect at severely low PSE (-1SD)	-0.020	.127	-0.270	0.230
Direct effect at moderately low PSE (Mean)	-0.080	0.091	-0.258	0.098
Direct effect at mildly low PSE (+1SD)	-0.140	0.115	-0.364	0.085

Note. Mediator = Pain Catastrophising; PSE = Pain Self-Efficacy; SD = Standard Deviation.

**Fig 2:**
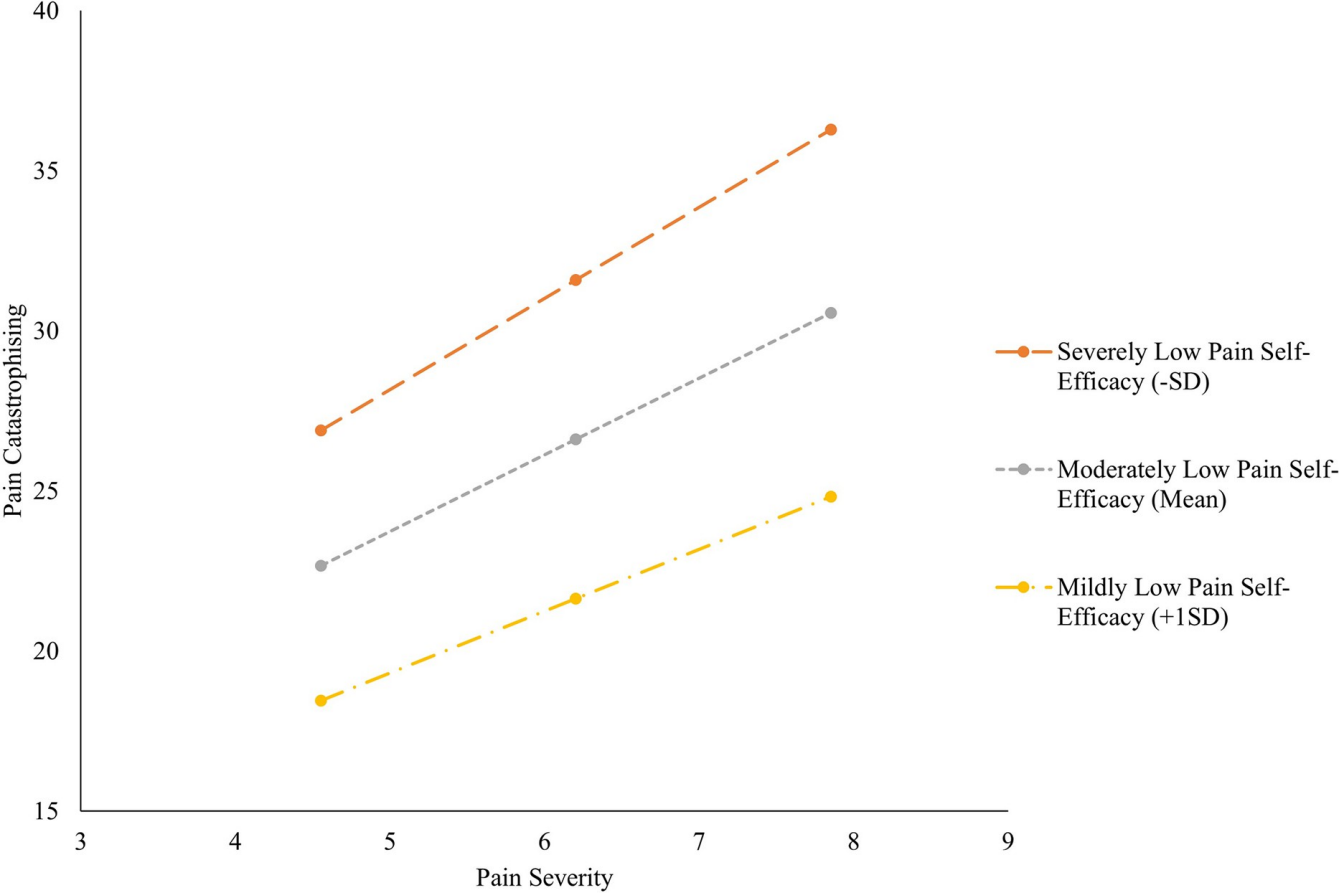
The Conditional Effects of Pain Self-Efficacy on the Relationship Between Pain Severity and Pain Catastrophising. *Note*. Variables are presented uncentred for illustrated purposes. Uncentred results did not significantly differ from centred results.

Both of these conditional indirect effects were then explored at different levels of pain self-efficacy. As illustrated in Fig 2, the effect for pain severity on pain catastrophising was β = 2.867, *CI* [2.298, 3.436], when pain self-efficacy was severely low (1SD below the mean), and β = 2.006, *CI* [1.472, 2.539] when pain self-efficacy was mildly low (1SD above the mean). The Johnson-Neyman technique was then utilised to examine the significance of this indirect effect at different values of the moderator. This showed that the magnitude of the relationship slightly attenuates as pain self-efficacy increases. Further, a significant transition point was shown at values of pain self-efficacy ≥ 38.97 (β = 1.106, *CI* [-.028, 2.237]), at which point the relationship between pain severity and pain catastrophising was non-significant. That is, once pain self-efficacy scores were approaching the minimally low (or normal; > 40) range, there was no association between pain severity and pain catastrophising.

**Fig 3:**
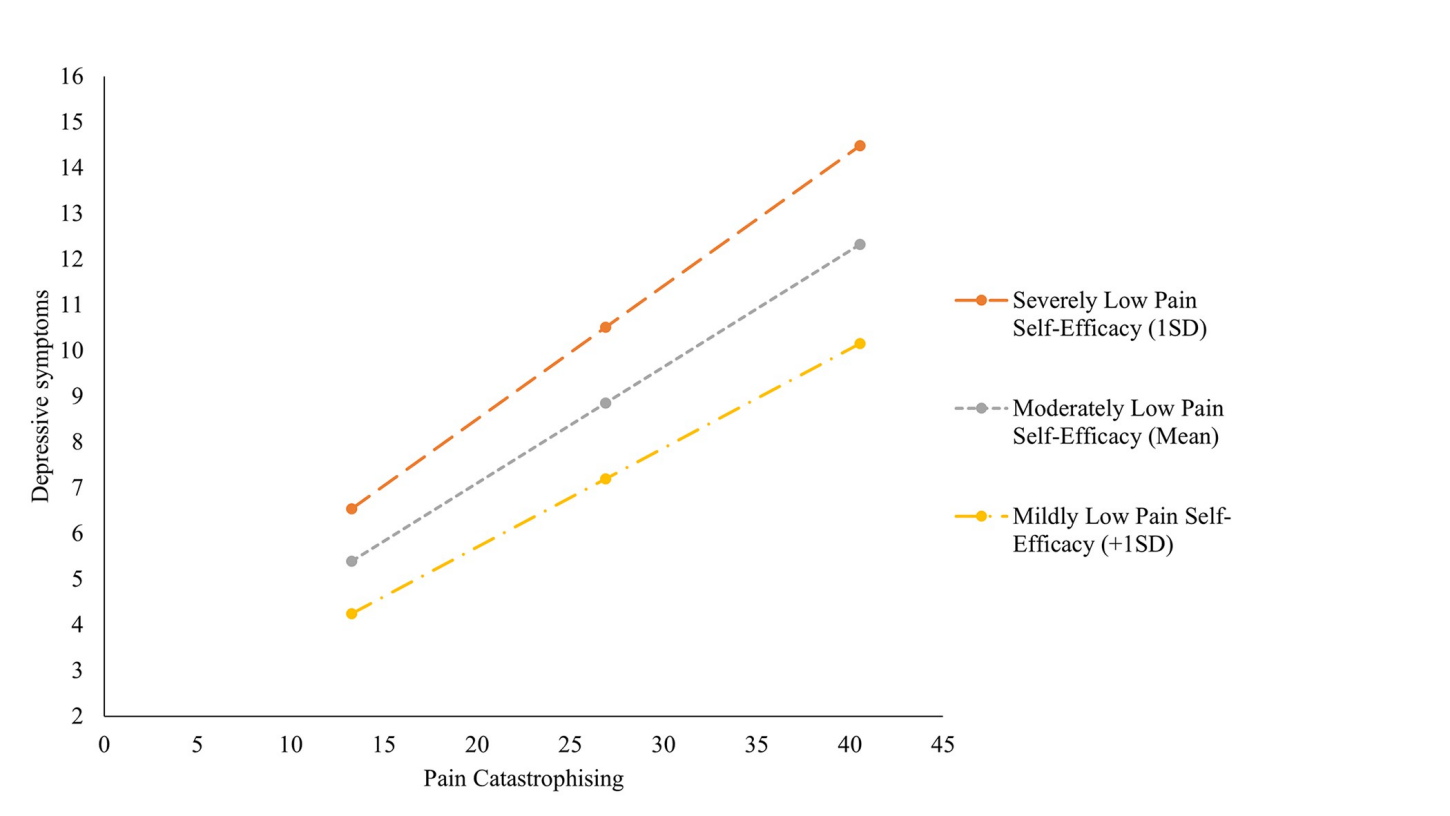
The conditional effects of pain self-efficacy on the relationship between pain catastrophising and depressive symptoms. *Note*. Variables are presented uncentred for illustrated purposes. Uncentred results did not significantly differ from centred results.

As illustrated in Fig 3, the moderating effect on the association between pain catastrophising and depressive symptoms remained significant at all levels of pain self-efficacy. Specifically, this effect was β = .291, *CI* [.261, .321] when pain self-efficacy was severely low (1SD below the mean), β = .234, *CI* [.231, .276] for moderatley low (mean) pain self-efficacy, and β = .216, *CI* [.185, .247] when pain self-efficacy was mildly low (1SD above the mean). This suggests that the magnitude of the relationship between pain catastrophising and depressive symptoms is slightly weakened as pain self-efficacy increases. Interestingly, changes in average depressive symptoms scores at low and high levels of pain self-efficacy reflect changes in the severity range of depressive symptoms, when pain catastrophising is high. Specifically, scores indicate that depressive symptoms are in the extremely severe range when pain self-efficacy is severely low and are in the moderate range when pain self-efficacy is mildly low. Changes in depression severity range at lower levels of pain catastrophising (e.g., in the mild to high range) are less pronounced.

## Discussion

The present study examined the role of pain catastrophising and pain self-efficacy in the relationship between pain severity and depression in an adult chronic pain clinical sample. The nature of these relationships was examined using a moderated mediation model that was expanded from an integrated cognitive model recently proposed by Cheng et al. [8]. In this current model, it is assumed that the relationship between pain severity and depressive symptoms is mediated by pain catastrophising, in addition to pain self-efficacy having a total moderation effect on all relationships. The hypothesis that pain catastrophising would mediate the relationship between pain severity and depressive symptoms was supported. This finding is consistent with previous research [8,10,12]. Additionally, pain self-efficacy was shown to slightly moderate the strength of this indirect association. Specifically, as levels of pain self-efficacy decreased, the effect of pain severity on the level of depression via pain catastrophising increased. This suggests that while pain catastrophising may be a significant risk factor for higher levels of depression in individuals with chronic pain, pain self-efficacy may play a significant role as a protective factor by influencing the strength of this relationship.

While research into the effects of cognitive factors on chronic pain outcomes like depression has been growing, limited studies have explored pain self-efficacy as a moderating variable. Pain self-efficacy is argued to be a psychological protective factor for individuals with chronic pain and therefore may be conceptualised as a moderating factor. Cheng et al. [8] offered a model that conceptualises pain self-efficacy as a moderating factor for individuals with chronic pain. However, in their model, it is assumed that pain self-efficacy only moderates the direct relationship between pain severity and depression as well as the first stage of the mediation between pain severity and pain catastrophising [8]. This current study extended this model by testing whether pain self-efficiacy influenced the magnitude of the relationships in all paths of the model. Specifically, it was hypothesised that pain self-efficacy would not only moderate the relationship between pain severity and pain catastrophising but would also moderate the relationship between pain catastrophising and depressive symptoms. Findings supported this hypothesis and indicate that even when individuals are engaging in pain catastrophic cognitions, pain self-efficacy may slightly reduce the strength of the relationship these cognitions have with depression. At higher levels of pain catastrophising, the influence of pain self-efficacy was further reflected in the change in depression severity range from extremely severe to moderate when pain self-efficacy was severely low and midly low, respectively. One potential interpretation of this interaction is that despite an individual engaging in catastrophic thinking, pain self-efficacy may still act as a protective factor against depressive symptoms. Specifically, by having higher pain self-efficacy beliefs in one’s ability to persist despite their pain, it would be expected that an individual would be more likely to be engaging in adaptive behavioural responses. As a result, these individuals may be less likely to engage in unhelpful behaviours such as inactivity or avoidance which are considered to contribute to the maintenance of depression [48].

The mediating role of pain catastrophising on the pain severity-depression relationship is consistent with previous research [8,10]. Also as noted, the moderating effect of pain self-efficacy on the mediation model was consistent with our expectations. Further, the direct assocation between pain severity and depression was fully mediated in the model, irrespective of the level of pain self-efficacy. Taken together, these finding support the notion that pain severity influences depression severity via pain catastrophising and the strength of indirect path varies as a function of pain self-efficacy. However, it appears pain self-efficacy may also attentuate the direct relationship between pain severity and depression given the main effect of pain self-efficay on depression (see Table 2). That is, pain self-efficacy and pain catastrophising appear to co-jointly account for the total effects of pain severity on depressive symptoms, which is consistent with research that supports a mediation role of pain self-efficacy [31,32]. This suggests that pain self-efficacy may not only operate as a pre-exisiting protective factor but may also be shaped by pain severity, and then in turn influence depressive symptoms. Thus, protecting and endering increased levels of pain self-efficacy may be particularly salient in chronic pain management.

Chronic pain and comorbid depression is a considerable health problem in Australia and is highly prevalent in the population [2]. Notably, this comorbidity has been shown to be associated with poorer treatment response and higher healthcare burden [2,5,6,19]. Currently, just over half of individuals report clinically significant improvement in depression following the completion of an episode of care in a pain management service [4]. This demonstrates the importance of continued research into the relevant contributing and protective factors and the nature of the relationships between factors in order to improve outcomes for those experiencing chronic pain. The results from the current study provide further insight into the nature of the relationships between these variables suggesting that while an individual’s level of pain catastrophising is a crucial factor in determining a person’s level of depressive symptoms, pain self-efficacy may act as an important protective factor. This may have important clinical implications including the manner in which measures are utilised in pain management services. Currently, measures used in this study are typically used as separate outcome measures under the model-of-care in clinical pain management settings [4,34]. However, demonstrated significant relationships between measures in this current study suggest they are better conceptualised within the context of each other. Given this, these measures may be more beneficially utilised to inform triage processes and treatment planning. Future directions for research may therefore include the evaluation of a changed model-of-care that trials the efficacy of measurement evaluation in pain management triaging, and service delivery. Evaluation during triage may allow clinicians to consider presenting levels of pain catastrophising and pain self-efficacy in informing initial treatment planning. For patients presenting with high levels of pain catastrophising and low pain self-efficacy, cognitive barriers may be larger to overcome during treatment. However, interventions that specifically target the crucial factor of pain catastrophising, in addition to having a key focus on increasing pain self-efficacy, may lead to a significant reduction in depression. Considering the recently reported variations in patient response to pain management interventions, it is essential that research continues to examine the efficacy of interventions in producing clinically significant change and improving patient outcomes [4].

A few limitations of this study are important to consider. First, while the data examined in this study appear reasonably consistent with nationally collected data via the ePPOC initiative, it would be beneficial to replicate this study in other Australian clinical samples to assess for similar relationships. For instance, future research could be performed with specific pain populations (eg: back pain, leg pain,neuropathic pain, post-surgical pain, musculoskeletal pain) to determine whether these findings generalise across contexts. Furthermore, while we did adjust for several important theoretical confounders, we were unable to adjust for additional potential confounders previously used in some of the literature, such as education level, marital status, employment status, number of pain sites and comorbid conditions, and sleep [8,9,16,27,29,49]. In line with the confounder effects in these studies, the true effects may be somewhat overestimated in our study. Adjusting for a broader range of co-variates would provide increased confidence in the validity of our findings. Also despite the study having a strong theoretical basis, it was not possible to test the temporal, reciprocal or the causative nature of the relationships depicted in the model. For instance, as noted by other researchers [50,51], depressive symptoms may have an influence on cognitive processes and functional outcomes. Similarly, as acknowledged by Cheng et al. [8] the mediation effects found in cross sectional research may differ from estimates found in longitudinal studies. Taken, together, future research is required to test the moderating and mediating roles of pain self-efficacy and pain catastrophising in multi-wave treatment/intervention studies where the temporal and reciprocal relationships and effect sizes can be further examined.

In summary, the findings from this current study provide insight into how the cognitive factors of pain catastrophising and pain self-efficacy may contribute to the level of depression severity in individuals with chronic pain. Specifically, findings from this study are consistent with previous research suggestingpain catastrophising mediates the relationship between pain severity and depressive symptoms. Further, the findings support the contention that pain self-efficacy may play a protective role by influencing the strength of therelationships between pain severity, pain catastrophising and depression symptoms. In addition to theoretical implications, findings from this study may have important clinical implications. This includes considering the roles of both pain catastrophising and pain self-efficacy in the maintaince of the relationship between chronic pain severity and depression. Consideration of these relationships in the planning and provision of treatment for individuals living with chronic pain may lead to improved outcomes.

## Supporting information

S1 Checklist (DOCX)
